# Comparative Efficacy of Pharmacological Interventions for Chronic Prostatitis/Chronic Pelvic Pain Syndrome: An Updated Systematic Review and Meta-Analysis of Randomized Controlled Trials

**DOI:** 10.3390/healthcare13222956

**Published:** 2025-11-18

**Authors:** Saad Alshahrani, Basem A. Fathi, Tamer A. Abouelgreed, Ashraf El-Metwally

**Affiliations:** 1 Department of Surgery, Division of Urology, College of Medicine, Prince Sattam bin Abdulaziz University, Al-Kharj 16278, Saudi Arabia; 2 Department of Urology, Faculty of Medicine, Al-Azhar University, Cairo 11884, Egypt; basemabdalla.8@azhar.edu.eg (B.A.F.); tamerali.8@azhar.edu.eg (T.A.A.); 3 College of Public Health and Health informatics, King Saud bin Abdulaziz University for Health Sciences, Riyadh 11481, Saudi Arabia; elmetwally.ashraf@outlook.com; 4 King Abdullah International Medical Research Center, Riyadh 12211, Saudi Arabia

**Keywords:** chronic prostatitis, chronic pelvic pain syndrome, pharmacological treatments, meta-analysis, NIH-CPSI

## Abstract

Background: Chronic prostatitis/chronic pelvic pain syndrome (CP/CPPS) is a prevalent and debilitating urological condition affecting approximately 2–10% of men globally, with a substantial impact on quality of life, productivity, and healthcare utilization. Despite the availability of multiple pharmacological options, their comparative efficacy remains uncertain. This meta-analysis evaluated the efficacy of pharmacological interventions for CP/CPPS based on the NIH-Chronic Prostatitis Symptom Index (NIH-CPSI). Methods: A systematic search of PubMed, Scopus, ScienceDirect, and Google Scholar was conducted from database inception to January 2025 for randomized controlled trials (RCTs) comparing pharmacological therapies with placebo. The primary outcome was a mean reduction in NIH-CPSI total score, with a clinically meaningful improvement defined as a ≥6-point reduction. Pooled mean differences (MDs) with 95% confidence intervals (CIs) were calculated using random-effects models, and risk of bias was assessed using the Cochrane Risk of Bias tool. The certainty of evidence was evaluated using the GRADE approach. Results: Alpha-blockers demonstrated the most consistent benefit (MD: −5.13; 95% CI: −6.87 to −3.39; Low certainty), followed by Traditional Chinese Medicine (TCM) (MD: −3.14; 95% CI: −5.38 to −0.90; Low certainty) and analgesics (MD: −2.47; 95% CI: −4.24 to −0.70; Low certainty). In contrast, antibiotics (MD: −2.45; 95% CI: −5.53 to 0.64; Very Low certainty), pollen extracts (MD: −2.56; 95% CI: −10.83 to 5.71; Very Low certainty), and other agents such as botulinum toxin A and anticonvulsants (MD: −6.94; 95% CI: −19.79 to 5.91; Very Low certainty) did not achieve statistical significance. The certainty for all interventions was downgraded from High due to risk of bias and substantial heterogeneity (*I*^2^ > 75%). Funnel plot asymmetry suggested potential publication bias; however, Egger’s test did not confirm statistical significance (*p* = 0.626). Conclusions: Among available pharmacological options, alpha-blockers and TCM provide the most reliable symptom improvement in men with CP/CPPS, while analgesics offer modest benefit. Antibiotics, pollen extracts, and other agents show inconsistent or non-significant effects. The high heterogeneity and generally low certainty of evidence reflect variability in study quality and populations, underscoring the need for rigorously designed, standardized future trials to guide patient-centered therapy selection.

## 1. Introduction

Chronic prostatitis/chronic pelvic pain syndrome (CP/CPPS) is a debilitating urological condition characterized by persistent pelvic pain, urinary dysfunction, and sexual symptoms, significantly impairing quality of life among affected men [1,2]. Globally, CP/CPPS affects an estimated 2–10% of men, representing a substantial public health concern due to its high prevalence, chronicity, and socioeconomic burden [3]. Sexual dysfunction is a common and impactful manifestation, with a pooled prevalence of 59% among 5333 participants, including erectile dysfunction in 34% and premature ejaculation in 35% [4].

The National Institutes of Health-Chronic Prostatitis Symptom Index (NIH-CPSI) remains the most widely used instrument to quantify symptom burden, with a reduction of ≥4–6 points generally regarded as clinically meaningful [5]. Over the past two decades, numerous pharmacological agents—ranging from alpha-blockers and antibiotics to anti-inflammatories, phytotherapeutics, and Traditional Chinese Medicine (TCM)—have been investigated [6]. However, their adoption varies considerably by region; Western practice often favors antibiotics and alpha-blockers, whereas TCM-based regimens are predominantly used in Asian settings, reflecting both divergent pathophysiological theories and cultural prescribing patterns [7,8].

Previous meta-analyses have examined pharmacological and non-pharmacological approaches to CP/CPPS, yet important gaps persist. For instance, Franco et al. (2019) conducted a comprehensive Cochrane review of pharmacological interventions, reporting low to very low certainty of evidence across most drug classes due to study limitations, inconsistency, and imprecision [9]. Wang et al. (2024) and Qin, Peipei et al. (2025) further explored acupuncture-based therapies, highlighting their potential benefits but also emphasizing patient-level variability in treatment response [10,11]. Despite these contributions, a contemporary synthesis that compares all major pharmacological categories within a unified analytical framework is lacking. The present meta-analysis addresses this gap by providing the largest and most up-to-date quantitative synthesis to 2025, encompassing randomized controlled trials (RCTs) across multiple drug classes and geographic regions. By integrating subgroup analyses based on study quality and setting, this work aims to clarify comparative efficacy, explore sources of heterogeneity, and assess the overall certainty of evidence using the GRADE framework. The objective of this meta-analysis is to evaluate and compare the effectiveness of major pharmacological interventions versus placebo in improving NIH-CPSI total scores among men with CP/CPPS.

## 2. Materials and Methods

### 2.1. Databases and Search Strategy

This systematic review and meta-analysis was conducted and reported in accordance with the Preferred Reporting Items for Systematic Reviews and Meta-Analyses (PRISMA) 2020 guidelines. A comprehensive literature search was conducted in PubMed, Scopus, and Science Direct to identify relevant RCTs. Google Scholar was used for supplementary citation chasing to ensure completeness of references, but not as a primary source. The search covered all studies from database inception up to 31 December 2024. The strategy combined controlled vocabulary and free-text terms, including: “chronic prostatitis”, “chronic pelvic pain syndrome”, “alpha blockers”, “analgesics”, “antibiotics”, “pollen”, “traditional Chinese medicine”, “anticonvulsants”, “botulinum toxin A”, and “randomized controlled trial”. Detailed search strategies used in each database are provided In Supplemental Table S1. Boolean operators were applied to refine combinations. Reference lists of included studies and relevant reviews were screened for additional eligible trials. Only full-text articles published in English were included.

### 2.2. Eligibility Criteria

Studies were included if they enrolled adult men diagnosed with chronic prostatitis/chronic pelvic pain syndrome (CP/CPPS) and evaluated pharmacological treatments such as alpha blockers (terazosin, doxazosin, phenoxybenzamine, tamsulosin, alfuzosin, silodosin), antibiotics (ciprofloxacin, levofloxacin), finasteride, anti-inflammatory agents (NSAIDs, corticosteroids, antileukotrienes, tiocolchicoside), phytotherapeutics (pollen extract, calendula–curcuma, Prolit Super Septo^®^, flavonoids, cranberries), Traditional Chinese Medicine (decoctions, capsules, suppositories), and other medications including botulinum toxin A, anticonvulsants, and 5-alpha reductase inhibitors. Grouping of “other medications” was justified on pragmatic grounds due to their limited representation in the literature; although mechanistically distinct, they were considered collectively to explore their potential role in symptom management. Inclusion of TCM was justified on methodological grounds, as these interventions have been rigorously evaluated in prior systematic reviews and RCTs using standardized formulations and comparable outcome measures (NIH-CPSI). Grouping them within pharmacological therapies ensured a comprehensive synthesis of all medication-based interventions for CP/CPPS. Eligible comparators consisted of placebo or inactive controls. For each included trial we extracted the NIH-CPSI total score as the primary outcome. While NIH-CPSI subdomain scores (pain, urinary symptoms, quality of life) and other QoL instruments were noted when reported, these measures were inconsistently and incompletely reported across studies; therefore, pooling of subdomains or separate QoL metrics was not feasible. The quantitative synthesis was consequently restricted to the NIH-CPSI total score, which provided the most robust and comparable outcome across trials. Only RCTs were considered for inclusion. Non-drug therapies were excluded to maintain focus on pharmacological efficacy and ensure methodological consistency.

### 2.3. Study Selection and Data Extraction

Titles and abstracts were independently screened by two reviewers. Full texts of potentially eligible studies were retrieved and assessed against inclusion criteria. Discrepancies were resolved by consensus or discussion with a third reviewer. For overlapping data or duplicate publications, the most comprehensive or recent dataset was included. Studies involving combination therapies were only included if the pharmacological effect could be isolated from co-interventions. Data were extracted using a standardized form, including study characteristics (author, year, country), participant demographics, intervention details (dose, duration), comparator, sample size, outcomes (mean, standard deviation), and follow-up duration.

### 2.4. Quality of Studies

The quality of the included studies was assessed using the Cochrane Risk of Bias (RoB) tool. This tool evaluates bias across several domains, including selection bias, performance bias, detection bias, attrition bias, reporting bias, and other biases. Each study was rated as having a low, high, or unclear risk of bias for each domain. The overall quality of evidence was summarized in a risk of bias table.

### 2.5. Certainty of Evidence (GRADE Assessment)

The certainty of evidence for each main comparison and outcome, measured by the NIH-CPSI Total scores, was systematically assessed using the GRADE (Grading of Recommendations Assessment, Development and Evaluation) approach [12]. The certainty of evidence was ultimately categorized as high, moderate, low, or very low. Given that all included studies were RCTs, the initial certainty for all outcomes was considered High. This initial rating was systematically downgraded across five domains where limitations were identified. Specifically, evidence was downgraded by one or two levels for Risk of Bias when a majority of the contributing studies were judged to have a high or unclear risk of bias, based on the findings of the Cochrane Risk of Bias tool. Downgrading also occurred for Inconsistency (Heterogeneity) when the statistical heterogeneity was substantial (>50%), indicating significant variability in treatment effects across studies. Further, the evidence was downgraded for Imprecision if the confidence interval (CI) was wide and crossed the line of no effect, or if the total number of participants was small, suggesting an inability to accurately estimate the true effect. The potential for Indirectness was assessed for relevance to the research question, and the risk of Publication Bias was evaluated through visual inspection of the funnel plot, though statistical non-significance on Egger’s test mitigated the need for downgrading in this domain. The final certainty of evidence ratings were then presented in a Summary of Findings table.

### 2.6. Statistical Analysis

Meta-analyses were performed using R software (version 4.2.0) with the *meta*, *metafor*, and *dmetar* packages. Pooled mean differences (MD) with 95% confidence intervals (CI) were calculated for continuous outcomes. Heterogeneity was quantified using Cochran’s Q and the *I*^2^ statistic, with thresholds of low (<25%), moderate (25–50%), and high (>50%) heterogeneity. A random-effects model (DerSimonian–Laird method) was employed to account for expected between-study variability. Clinical effectiveness was defined as a reduction of ≥6 points in NIH-CPSI, representing a meaningful improvement in symptoms. Subgroup analyses were conducted to explore potential sources of heterogeneity. For alpha-blockers, which had sufficient data from more than ten RCTs, additional subgroup analyses were performed by risk of bias (high, low, unclear) and study setting (Asian vs. Western) to assess the robustness and consistency of findings across methodological and regional contexts. These analyses are presented in Section 3 and Supplementary Figures. Funnel plot symmetry and Egger’s test were used to evaluate potential publication bias.

## 3. Results

### 3.1. Screening and Flow of Studies

**Figure 1:**
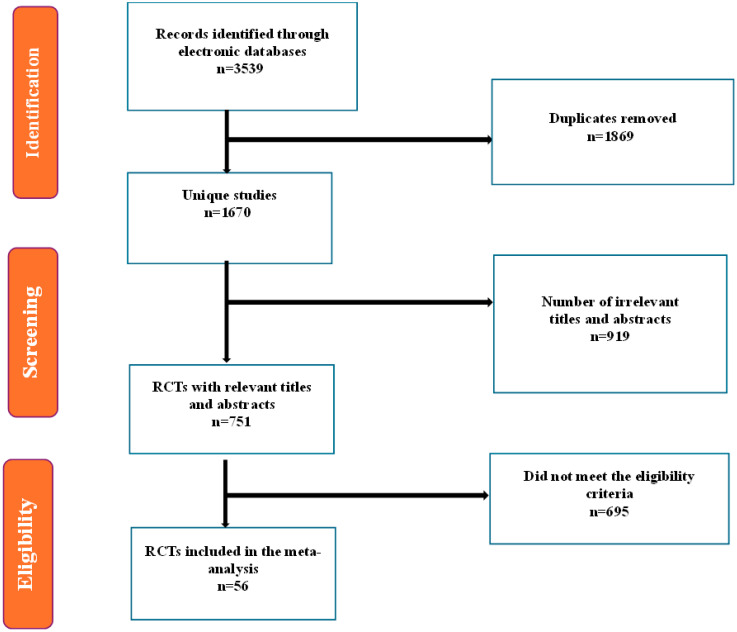
PRISMA flow diagram showing the identification, screening, eligibility assessment, and inclusion of studies. The literature search was conducted up to 31 December 2024.

A comprehensive search initially identified 3539 records from various databases. After the exclusion of 1869 duplicates, 1670 articles remained for title and abstract screening. Of these, 919 articles were excluded for not meeting the inclusion criteria at the abstract and title screening stage. Subsequently, 751 full-text articles were reviewed, and 695 were excluded for reasons such as inappropriate study design, irrelevant outcomes, or unsuitable populations. Ultimately, 56 records of RCTs were included in the qualitative synthesis and meta-analysis (Figure 1).

Supplemental Table S2 reveals the characteristics of RCTs included in systematic review and meta-analysis. A total of 56 studies were included in this systematic review and meta-analysis, spanning a period from 1999 to 2019. Of these, 13 studies were conducted between 1999 and 2005, 23 studies between 2006 and 2010, 14 studies from 2011 to 2015, and 6 studies from 2016 to 2019. This distribution reflects the evolving interest and continued research efforts in the field of CP/CPPS treatment over the past two decades.

#### Characteristics of Eligible Studies

As shown in Table 1, geographically, the studies were conducted across various regions, indicating a widespread interest in CP/CPPS. Specifically, there was one study each from Turkey, Iran, Bosnia and Herzegovina, Finland, Germany, and Italy; two studies each from Canada and the Republic of Korea; three studies from Russia; 21 studies from China; four studies across Europe; three studies from North America; four multi-country studies; seven studies from South Korea; and two studies each from Turkey and the USA (Table 1). This diverse geographical representation underscores the global significance of CP/CPPS and the efforts to identify effective treatment strategies.

Regarding the interventions, the analysis included various pharmacological treatments as illustrated in Table 1. Two studies assessed the efficacy of 5-alpha reductase inhibitors, 21 studies tested alpha-blockers, seven evaluated analgesics, five studies investigated antibiotics, and one study evaluated anticonvulsant. Additionally, two studies explored the effects of Botulinum toxin A, nine studies examined pollen extract, and another nine studies focused on Traditional Chinese Medicine (TCM). This wide range of interventions highlights the diverse approaches taken to managing CP/CPPS and provides a comprehensive overview of their relative efficacy.

### 3.2. Effect of Alpha Blockers vs. Placebo

**Figure 2:**
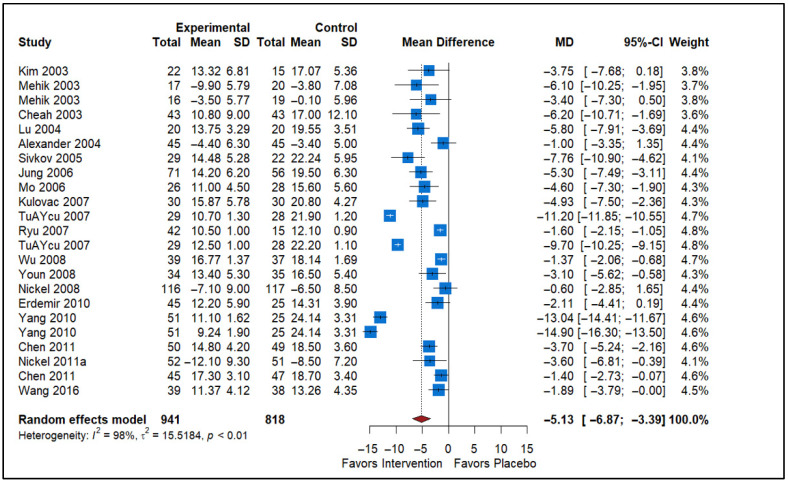
Forest plot showing the pooled effect of alpha-blockers on NIH-CPSI total scores in men with chronic prostatitis/chronic pelvic pain syndrome (CP/CPPS). Negative MD indicate greater symptom improvement compared with placebo (Nickel, 2008 [7], Cheah, 2003 [13], Erdemir, 2010 [14], Jung, 2006 [15], Wang, 2016 [16], Yang 2010 [17], Kulovac, 2007 [18], TuAYcu, 2007 [19], Wu, 2008 [20], Youn, 2008 [21], Lü,2004 [22], Alexander, 2004 [23], Chen, 2011 [24], Kim, 2003 [25], Mehik, 2003 [26], Mo, 2006 [27], Ryu, 2007 [28], Nickel, 2011a [29], Sivkov, 2005 [30]).

The forest plot provides a summary of the pooled effects of alpha-blockers compared to placebo on the NIH-CPSI Total scores for Chronic Prostatitis (Figure 2). The studies included in the analysis show a range of MD in the effect of alpha-blockers on the NIH-CPSI Total scores, with values ranging from −16.30 to 0.50, indicating variability in the effects across different studies. The mean difference column reflects the difference between the experimental and control (placebo) groups, with the negative values favoring the intervention (alpha-blockers). The pooled MD is −5.13, with a 95% confidence interval (CI) of −6.87 to −3.39, suggesting that alpha-blockers are statistically significantly more effective than placebo in reducing the NIH-CPSI Total scores for Chronic Prostatitis. The diamond at the bottom of the plot, which represents the overall pooled estimate, lies to the left of the vertical line, indicating a favoring of the intervention over the placebo. The heterogeneity statistic (*I*^2^ = 98%) suggests substantial variability across studies, implying that factors other than the treatment itself may contribute to the differences in outcomes. This high heterogeneity, reflected by the tau^2^ statistic of 15.5184, suggests that the studies may differ significantly in their methodologies or patient populations. Despite this heterogeneity, the result supports the efficacy of alpha-blockers in reducing symptoms of Chronic Prostatitis as measured by the NIH-CPSI Total scores when compared to a placebo.

### 3.3. Effect of Analgesics vs. Placebo

**Figure 3:**
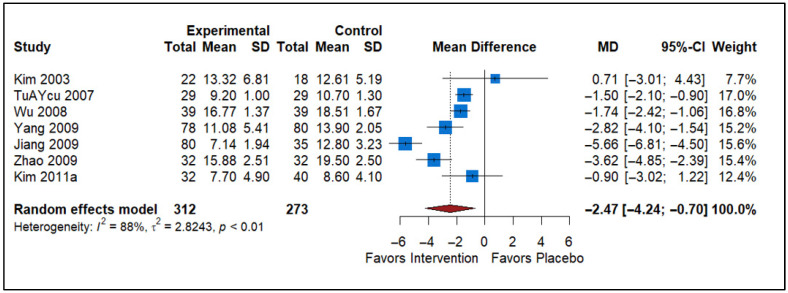
Forest plot summarizing the impact of analgesics on NIH-CPSI total scores among CP/CPPS patients. The plot demonstrates whether pain-relieving agents provide clinically meaningful reductions compared to placebo (Kim [25], TuAYcu [19], Wu [20], Yang [33], Jiang [34], Zhao [35], Kim [31]).

The forest plot includes seven studies comparing the effects of analgesics versus placebo on the NIH-CPSI Total scores for Chronic Prostatitis (Figure 3). The individual studies (n = 7) show variability in their results, with the MD ranging from −5.66 to 0.71. A negative MD indicates that analgesics are more effective than placebo in reducing the NIH-CPSI scores, while a positive MD favors placebo. The pooled MD is −2.47, with a 95% confidence interval (CI) of −4.24 to −0.70, indicating a statistically significant reduction in symptoms with analgesics compared to placebo. However, the heterogeneity statistic (*I*^2^ = 88%) indicates substantial variability among the studies, suggesting differences in study design, patient populations, or intervention protocols. The tau^2^ value of 2.8243 further supports the presence of high variability. Overall, while the data supports the efficacy of analgesics in reducing NIH-CPSI scores, the high heterogeneity warrants cautious interpretation of the pooled result and consideration of study-level differences.

### 3.4. Effect of Antibiotics vs. Placebo

**Figure 4:**
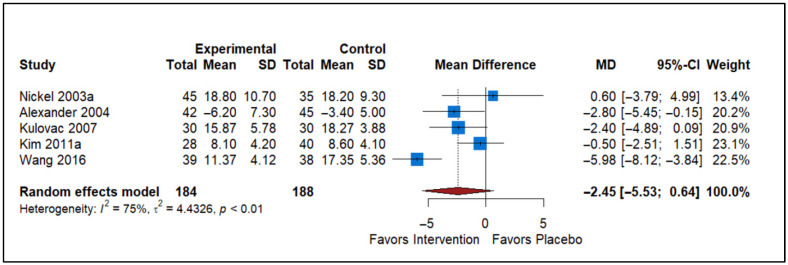
Forest plot illustrating the effect of antibiotics on NIH-CPSI total scores in CP/CPPS. The pooled estimates reflect the extent to which antimicrobial therapy improves overall symptom burden beyond placebo (Nickel 2003a [32], Alexander 2004 [23], Kulovac 2007 [18], Kim 2011a [31], and Wang 2016 [16]).

The forest plot includes data from five studies comparing the effects of antibiotics versus a placebo on the NIH-CPSI Total scores for Chronic Prostatitis (Figure 4). The studies included in the analysis are Nickel 2003a [32], Alexander 2004 [23], Kulovac 2007 [18], Kim 2011a [31], and Wang 2016 [16]. In the experimental group (Antibiotics), there were a total of 184 participants, while the control group had 188 participants. The plot displays the MD and 95% confidence intervals (CI) for each study. The overall MD is −2.45, with a 95% confidence interval ranging from −5.53 to 0.64, indicating a statistically non-significant reduction in symptoms with antibiotics compared to placebo. The heterogeneity statistics indicate substantial variability among the studies (*I*^2^ = 75%, τ^2^ = 4.4326, *p* < 0.01). Although there is a general trend favoring the intervention, the wide confidence interval and high heterogeneity highlight variability among the study results. This analysis suggests that while the intervention may be beneficial, the inconsistency among the study outcomes warrants further investigation.

### 3.5. Effect of Pollen Extract vs. Placebo

**Figure 5:**
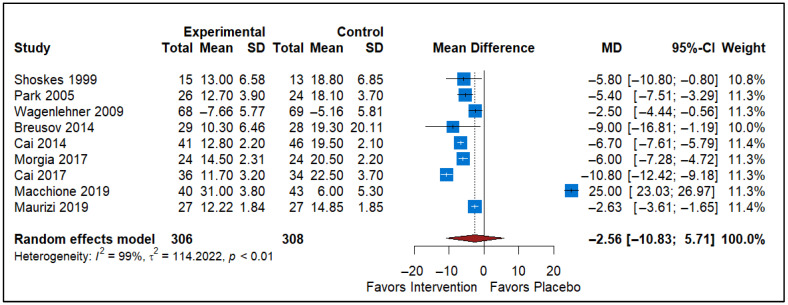
Forest plot depicting the efficacy of pollen extract compared with placebo on NIH-CPSI total scores, highlighting the potential role of phytotherapeutic agents in reducing CP/CPPS symptoms (Shoskes 1999 [46], Park 2005 [47], Wagenlehner 2009 [43], Breusov 2014 [44], Cai 2014 [51], Morgia 2017 [45], Cai 2017 [48], Macchione 2019 [49], and Maurizi 2019 [50]).

The forest plot includes data from nine studies comparing the effects of Pollen versus a placebo on a specific outcome (Figure 5). The studies included Shoskes 1999 [48], Park 2005 [49], Wagenlehner 2009 [45], Breusov 2014 [46], Cai 2014 [53], Morgia 2017 [47], Cai 2017 [50], Macchione 2019 [51], and Maurizi 2019 [52]. The analysis comprised a total of 707 participants in the experimental group and 706 participants in the control group. The MD for the overall effect is −2.56, with a 95% confidence interval ranging from −10.83 to 5.71, indicating that the results are not statistically significant since the confidence interval crosses zero. The heterogeneity statistics show a high level of variability among the included studies (*I*^2^ = 99%, τ^2^ = 114.2022, *p* < 0.01). Despite the overall trend favoring Pollen, the substantial heterogeneity and non-significant results suggest that the intervention’s effectiveness is inconclusive.

### 3.6. Effect of Traditional Chinese Medicine vs. Placebo

**Figure 6:**
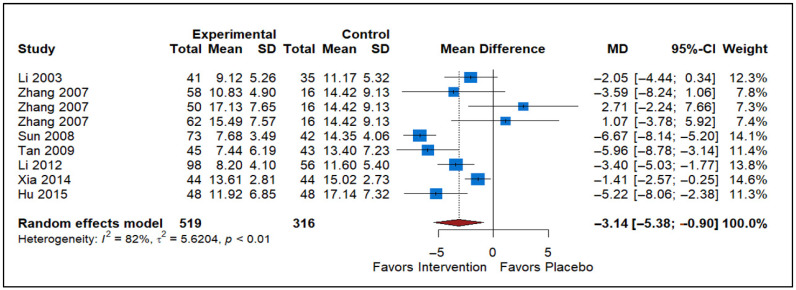
Forest plot evaluating Traditional Chinese Medicine interventions on NIH-CPSI total scores by synthesizing the evidence from international RCTs to assess their contribution to symptom relief (Li 2003 [38], Zhang 2007 (three entries) [43], Sun 2008 [41], Tan 2009 [39], Li 2012 [40], Xia 2014 [42], and Hu 2015 [44]).

The forest plot includes data from nine studies comparing the effects of Traditional Chinese Medicine (TCM) versus a placebo on a specific outcome (Figure 6). The studies included are Li 2003 [38], Zhang 2007 (three entries) [43], Sun 2008 [41], Tan 2009 [39], Li 2012 [40], Xia 2014 [42], and Hu 2015 [44]. The analysis comprises a total of 707 participants in the experimental group and 706 participants in the control group. The MD for the overall effect is −3.14, with a 95% confidence interval ranging from −5.38 to −0.90, indicating that the results are statistically significant in favor of TCM. The heterogeneity statistics show a high level of variability among the included studies (*I*^2^ = 82%, τ^2^ = 5.6204, *p* < 0.01). Despite the high heterogeneity, the overall effect suggests that Traditional Chinese Medicine has a beneficial impact in reducing the prostatitis symptoms compared to the placebo.

### 3.7. Effect of Other Medicines vs. Placebo

**Figure 7:**
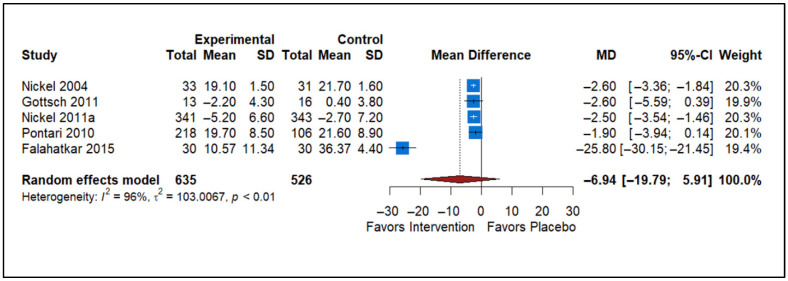
Forest plot showing the effect of “other medications”, including botulinum toxin A, anticonvulsants, and 5-alpha reductase inhibitors, on NIH-CPSI total scores in CP/CPPS (Nickel 2004 [55], Gottsch 2011 [37], Nickel 2011a [29], Pontari 2010 [56], and Falahatkar 2015 [36]).

The forest plot includes data from five studies comparing the effects of various medications (Botulinum Toxin A, anticonvulsants, and 5-alpha reductase inhibitors) versus a placebo on prostatitis symptoms (Figure 7). The studies included Nickel 2004 [55], Gottsch 2011 [37], Nickel 2011a [29], Pontari 2010 [56], and Falahatkar 2015 [36]. The analysis comprises a total of 635 participants in the experimental group and 526 participants in the control group. The overall MD is −6.94, with a 95% confidence interval ranging from −19.79 to 5.91, indicating that the results are not statistically significant since the confidence interval crosses zero. The heterogeneity statistics show significant variability among the included studies (*I*^2^ = 96%, τ^2^ = 103.0067, *p* < 0.01). Despite the overall trend favoring the medications, the substantial heterogeneity and non-significant results suggest that the effectiveness of these interventions is inconclusive.

### 3.8. Publication Bias

**Figure 8:**
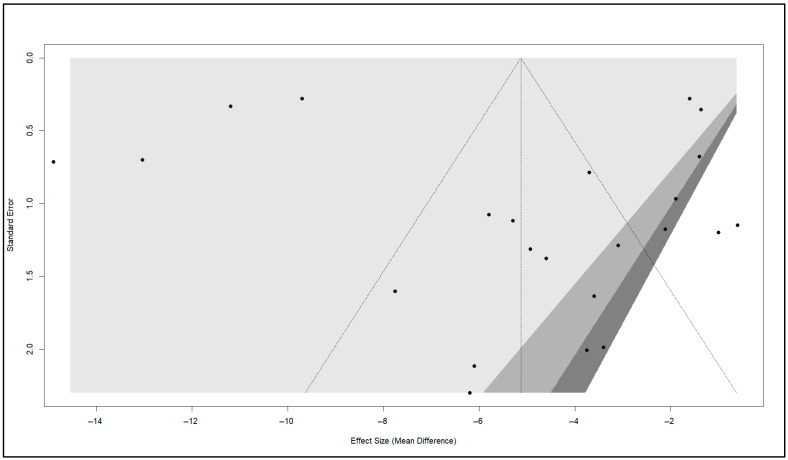
Funnel plot assessing publication bias across included RCTs. Symmetry of the plot suggests absence of small-study effects, whereas asymmetry would indicate possible bias in the published evidence base.

The funnel plot for the management of chronic prostatitis reveals some degree of asymmetry, with a higher concentration of studies on one side of the plot, particularly around smaller or larger effect sizes (Figure 8). This asymmetry may suggest the possibility of publication bias or heterogeneity in the included studies. The contour-enhanced funnel plot provides additional context by incorporating significance levels (0.9, 0.95, and 0.99), represented by progressively darker shaded regions. Studies falling outside the darker shaded areas are statistically significant at higher confidence levels, while those within these regions are less significant. This visualization underscores the tendency for published studies to cluster in areas of statistical significance, reflecting the potential for non-significant studies to remain unpublished or excluded. Additionally, small-study effects are evident, as studies with larger standard errors (positioned lower on the plot) demonstrate greater variability in effect sizes. This variability often leads to exaggerated or extreme effect sizes in smaller studies, further contributing to the asymmetry observed in the funnel plot.

However, Egger’s regression test of funnel plot asymmetry does not support the presence of significant publication bias (t = 0.49, df = 21, *p*-value = 0.6260). The intercept (−6.6473, SE = 1.6530) and bias estimate (1.3085, SE = 2.6458) indicate no substantial deviation from symmetry. Additionally, the multiplicative residual heterogeneity variance (tau^2^ = 58.1829) highlights substantial variability in effect sizes, which may also explain the asymmetry seen in the plot.

### 3.9. Risk of Bias: Quality of Studies

Table 1 presents the risk of bias assessment, detailing the quality of the included studies. The assessment of study quality using the Cochrane Risk of Bias tool revealed varied levels of methodological rigor across the 56 RCTs included in this meta-analysis. Specifically, 12 RCTs were identified as having a high risk of bias, primarily due to issues such as inadequate blinding, incomplete outcome data, or selective reporting. These limitations may affect the reliability and validity of their findings. Conversely, 16 RCTs were assessed to have a low risk of bias, indicating that these studies generally adhered to robust methodological standards, including proper randomization, blinding, and comprehensive reporting of outcomes. The high-quality evidence from these trials enhances the overall credibility of the meta-analysis results. The remaining 28 RCTs were categorized as having an unclear risk of bias. This uncertainty often arose from insufficient reporting of key methodological details, making it challenging to fully ascertain the potential for bias in these studies. While these trials contribute valuable data, the ambiguity regarding their methodological rigor warrants cautious interpretation of their findings.

### 3.10. Certainty of Evidence (GRADE Assessment)

For interventions where the overall effect was statistically non-significant, such as Pollen Extract vs. Placebo (MD = −2.56), the evidence was downgraded to Very Low due to Risk of Bias, extreme Inconsistency (*I*^2^ = 99%), and Imprecision (non-significant result with a very wide 95% CI. Similar downgrades were applied to Other Medications vs. Placebo (e.g., Botulinum Toxin A, anticonvulsants), resulting in a Very Low certainty rating. The certainty for Traditional Chinese Medicine (TCM) vs. Placebo was downgraded two levels to Low due to Risk of Bias and Inconsistency (*I*^2^ = 82%). The domain of Publication Bias did not lead to downgrading, as the Egger’s test was non-significant, despite visual asymmetry in the funnel plot, which was largely explained by high heterogeneity.

The certainty of evidence for each pharmacological intervention compared to placebo, as assessed by the GRADE approach, is summarized in Table 2 [The Summary of Findings Table]. The certainty for all comparisons was initially set at High due to the inclusion of only RCTs but was systematically downgraded due to limitations in the domains of Risk of Bias and Inconsistency (Heterogeneity). For Alpha-blockers vs. Placebo, the certainty of evidence was downgraded two levels to Low. One level was downgraded due to Risk of Bias (12 of 56 total studies had high risk, and 28 were unclear). An additional level was downgraded for Inconsistency, given the substantial and very high heterogeneity (*I*^2^ = 98%) and widely varying MDs across studies. For Analgesics vs. Placebo, the certainty was downgraded to two levels to Low, one for Risk of Bias and a second for Inconsistency (*I*^2^ = 88%). For Antibiotics vs. Placebo, the certainty was downgraded three levels to Very Low. Downgrades were applied for Risk of Bias, Inconsistency (*I*^2^ = 75%)), and Imprecision (the 95% CIs) crossed the line of no effect, and the total participant count was low.

### 3.11. Subgroup Analysis

A subgroup analysis was performed to examine the effect of alpha-blockers on NIH-CPSI total scores in men with CP/CPPS, stratified by the Risk of Bias (High, Low and Unclear) of the included studies. Subgroup analysis (Supplemental Figure S1) revealed that the largest and statistically significant treatment effect was seen in the studies with unclear risk (n = 13, MD: −6.69; 95% CI: −9.38 to −4.00, *I*^2^ = 98.9%). The high ROB subgroup also demonstrated a large reduction in pain score (n = 3, MD: −4.29; 95% CI: −9.14 to 0.55) with moderate heterogeneity (*I*^2^ = 64.6%). In contrast, the studies with Low ROB showed a smaller but statistically significant reduction (n = 7, MD: −1.76; 95% CI: −2.95 to −0.57), with lower heterogeneity *I*^2^ = 23.8%). The test for subgroup differences was statistically significant (*p* = 0.0003), suggesting that the effect of alpha-blockers on NIH-CPSI total scores is significantly influenced by the risk of bias in the primary studies.

A second subgroup analysis was performed to examine the effect of alpha-blockers on NIH-CPSI total scores in men with CP/CPPS, stratified by the study setting (Western vs. Asian countries). The subgroup analysis (Supplemental Figure S2) stratified by study setting (Western vs. Asian) showed no statistically significant difference in the effect of alpha-blockers on NIH-CPSI total scores (*p* = 0.263). Alpha-blockers demonstrated a significant reduction in scores in both the Asian setting n = 17, MD = −5.53, 95% CI = [−7.75, −3.32]) and the Western setting (MD = −3.76, 95% CI = [−6.81, −0.71]), with a numerically larger effect size observed in Asian studies. However, both subgroups were characterized by very high levels of heterogeneity (Asian setting subgroup *I*^2^ = 98.7%, and the Western setting *I*^2^ = 75.1%. This suggests that while the treatment effect appears consistent across geographic settings, the high variability in results within each subgroup indicates that other underlying differences among the trials are contributing substantially to the observed heterogeneity.

## 4. Discussion

This systematic review and meta-analysis synthesized evidence from 56 randomized RCTs evaluating pharmacological treatments for CP/CPPS. Quantitatively, alpha-blockers reduced NIH-CPSI scores by approximately 5 points compared with placebo, approximating the minimal clinically important difference (MCID) of 4–6 points, indicating meaningful symptom improvement. Analgesics and TCM also produced significant reductions in scores, though did not exceed MCID. Finally, antibiotics, pollen extract, and other pharmacologic agents demonstrated limited or inconsistent efficacy. These findings provide updated and comprehensive comparative evidence across pharmacologic modalities for CP/CPPS management.

Alpha-blockers emerged as the most consistently effective treatment, likely due to their dual action in relaxing prostatic smooth muscle and attenuating sympathetic overactivity, which alleviates both urinary and pain symptoms [57]. Increasing evidence suggests additional anti-inflammatory properties mediated by cytokine modulation, potentially explaining sustained symptom improvement beyond urodynamic effects [58]. These findings align with previous systematic reviews demonstrating similar trends [9,59,60], confirming alpha-blockers as a key first-line pharmacological option for CP/CPPS.

Analgesics provided moderate but clinically relevant pain relief, supporting their role as adjunctive rather than standalone therapy [61,62]. The moderate effect size observed here corroborates previous reviews [9,59,60], yet the inclusion of a larger number of RCTs in the present analysis strengthens the comparative evidence base. Because CP/CPPS pain often involves neuropathic and inflammatory mechanisms, future trials should explore multimodal regimens combining analgesics with agents targeting neuroimmune or central sensitization pathways.

TCM demonstrated consistent symptom reductions across multiple standardized formulations; however, their effect did not exceed MCID as supported by prior review [9]. Mechanistically, these benefits likely result from anti-inflammatory, antioxidant, and immune-modulating effects, rather than traditional philosophical concepts [63,64]. Experimental data indicate that active herbal constituents improve microcirculation and reduce oxidative stress, thereby facilitating symptom improvement [65,66]. TCM’s multi-target approach may offer unique advantages by addressing pain, urinary dysfunction, and quality of life concurrently. These findings align with previous meta-analyses showing similar efficacy trends [67]. Hence, while formulation standardization and trial quality remain limitations, the evidence supports TCM as a complementary pharmacologic strategy where conventional therapies are insufficient [67].

The current findings are broadly concordant with Franco et al. (2019), who reported low- to very low-quality evidence that alpha-blockers, antibiotics, anti-inflammatories, and TCM reduce prostatitis symptoms without significant adverse events [9]. Supporting this, Deng et al. (2020) conducted a systematic review and meta-analysis of six RCTs involving 450 patients and found that α-adrenergic receptor blockers significantly improved NIH-CPSI total scores [68]. However, Mishra et al. (2007) concluded that the published literature is insufficient to establish the efficacy of alpha-blockers for type III prostatitis with certainty and highlighted the need for future studies incorporating standardized data collection and reporting, with health-related quality of life as a key endpoint [69].

Our synthesis incorporates more recent data, expanding to 2025, and provides stronger pooled estimates across pharmacologic classes. In contrast to Franco et al. (2019), antibiotics, anti-inflammatory drugs, and other agents (e.g., anticonvulsants, 5-ARIs, botulinum toxin A) showed inconsistent or minimal benefit. This likely reflects the noninfectious pathophysiology of most CP/CPPS cases, small sample sizes, and heterogeneity in drug regimens. These findings further support the avoidance of empirical antibiotic therapy and highlight the need for precision-based treatment selection. Together, these results underscore the importance of well-designed, multicenter RCTs to generate more definitive, high-quality evidence for guiding CP/CPPS management.

### Strengths and Limitations

A significant strength of this meta-analysis is the comprehensive inclusion of a wide range of pharmacological treatments across multiple randomized controlled trials (RCTs). This allows for a robust synthesis of evidence and provides a clearer picture of the relative efficacy of different treatments. The use of the Cochrane Risk of Bias tool adds to the credibility of the findings by systematically assessing the quality of the included studies. Additionally, the reliance on the NIH-CPSI score as the primary outcome measure ensures consistency across the included studies. The NIH-CPSI is a widely accepted and validated tool for assessing the symptoms and quality of life in patients with CP/CPPS, providing a standardized method to compare results.

There are several limitations to consider in this review. High heterogeneity was observed across the included studies. To account for this, we applied a random-effects model, which considers both within-study and between-study variability and provides more conservative pooled estimates. Nevertheless, given the high heterogeneity, confidence in the pooled estimates should be interpreted with caution. Several factors may contribute to the observed heterogeneity. First, the included studies investigated a range of intervention types, including alpha-blockers, TCM, and other pharmacologic agents, each with different mechanisms of action and expected effect sizes. To minimize confounding, we conducted separate analyses for each intervention category rather than pooling them. Regional publication patterns, particularly for TCM, may have introduced cultural or language-related publication bias, as many trials were published in regional or non-indexed journals. Second, variation in follow-up duration across studies introduced further heterogeneity. Most trials had relatively short follow-up periods (<3 months), limiting evaluation of long-term outcomes or symptom recurrence. Moreover, adverse event reporting was inconsistent across RCTs, precluding a comprehensive safety assessment. Third, differences in methodological quality, study design, and participant characteristics (such as baseline severity and duration of symptoms) may also explain variability in effect estimates. Our subgroup analyses by ROB and study setting support the hypothesis that methodological rigor and regional differences contribute meaningfully to heterogeneity. However, the limited number of studies in other drug categories restricted the statistical power to perform subgroup analysis for all interventions. Finally, inconsistencies in the reporting of adverse events further limited the ability to fully assess risk–benefit profiles. Overall, the high heterogeneity likely reflects genuine differences in study populations and designs rather than random error, emphasizing the importance of cautious interpretation and the need for more standardized and methodologically rigorous trials. While contour-enhanced funnel plots suggested potential asymmetry and clustering of statistically significant studies, Egger’s regression test did not provide statistical evidence of publication bias. The observed asymmetry may reflect residual heterogeneity, methodological differences, or small-study effects rather than publication bias. The risk of bias assessment highlights variability in study quality, emphasizing the need to consider methodological differences when interpreting results. Additionally, although GRADE assessments were performed, the review was not prospectively registered in PROSPERO, which may limit transparency. Despite these limitations, this review provides the largest and most current synthesis to date of pharmacologic therapies for CP/CPPS, integrating recent evidence up to 2025. The findings highlight promising directions for future research, emphasizing the need for long-term, multicenter RCTs with standardized outcomes, particularly those comparing combination regimens and exploring biological mechanisms underlying symptom persistence.

## 5. Conclusions and Clinical Relevance

The findings of meta-analysis demonstrate that alpha-blockers and TCM provide the most consistent and clinically meaningful symptom relief for patients with CP/CPPS, while analgesics offer supportive value in alleviating pain. In contrast, antibiotics and other pharmacologic agents show inconsistent or limited benefits, reflecting variability in patient characteristics and study designs. These findings underscore the importance of individualized, phenotype-based management, where treatment is guided by dominant symptom patterns, pathophysiological mechanisms, and patient preferences rather than a uniform therapeutic approach. Future research should prioritize standardized, multicenter randomized controlled trials with longer follow-up durations and harm reporting to better define long-term efficacy, safety, and subgroup-specific treatment responses.

## Figures and Tables

**Table 1:** Quality of randomized controlled trials included in the systematic review and meta-analysis (n = 56).

Study	Year	Country	Risk of Bias
Cheah 2003 [13]	2004	Malaysia and United States of America	Unclear risk
Erdemir 2010 [14]	2010	Turkey	High risk
Jung 2006 [15]	2006	South Korea	Unclear risk
Wang 2016 [16]	2016	China	Low risk
Yang 2010 [17]	2010	China	Unclear risk
Kulovac 2007 [18]	2007	Bosnia and Herzegovina	High risk
TuAYcu 2007 [19]	2007	Turkey	Unclear risk
Wu 2008 [20]	2008	China	Unclear risk
Youn 2008 [21]	2008	South Korea	Unclear risk
Lu 2004 [22]	2004	China	High risk
Alexander 2004 [23]	2004	United States of America and Canada	Low risk
Chen 2011 [24]	2011	China	Low risk
Kim 2003 [25]	2003	South Korea	Unclear risk
Yang 2010 [17]	2010	China	Unclear risk
Mehik 2003 [26]	2003	Finland	Low risk
Mo 2006 [27]	2006	South Korea	Unclear risk
Nickel 2008 [7]	2008	United States of America, Canada and Malaysia	Low risk
Ryu 2007 [28]	2007	South Korea	Unclear risk
Nickel 2011a [29]	2011	Canada	Low risk
Sivkov 2005 [30]	2005	Russia	Unclear risk
Chen 2011 [24]	2011	China	Low risk
Alexander 2004 [23]	2004	United States of America and Canada	Low risk
Kim 2011a [31]	2011	Republic of Korea	High risk
Kulovac 2007 [18]	2007	Bosnia and Herzegovina	High risk
Nickel 2003a [32]	2003	Canada	Low risk
Wang 2016 [16]	2016	China	Low risk
Yang 2009 [33]	2009	China	Unclear risk
Jiang 2009 [34]	2009	China	Unclear risk
Kim 2003 [25]	2003	South Korea	Unclear risk
Kim 2011a [31]	2011	Republic of Korea	High risk
Wu 2008 [20]	2008	China	Unclear risk
Zhao 2009 [35]	2009	China	Unclear risk
TuAYcu 2007 [19]	2007	Turkey	Unclear risk
Falahatkar 2015 [36]	2015	Iran	Low risk
Gottsch 2011 [37]	2011	United States of America	High risk
Li 2003 [38]	2003	China	Unclear risk
Tan 2009 [39]	2009	China	High risk
Li 2012 [40]	2012	China	High risk
Sun 2008 [41]	2008	China	Unclear risk
Xia 2014 [42]	2014	China	Unclear risk
Zhang 2007 [43]	2007	China	High risk
Hu 2015 [44]	2015	China	Low risk
Zhang 2007 [43]	2007	China	Low risk
Zhang 2007 [43]	2007	China	Low risk
Wagenlehner 2009 [45]	2009	Germany	High risk
Breusov 2014 [46]	2014	Russia	Unclear risk
Morgia 2017 [47]	2017	Italy	Unclear risk
Shoskes 1999 [48]	1999	United States of America	Unclear risk
Park 2005 [49]	2005	South Korea	High risk
Cai 2017 [50]	2017	Europe	Unclear risk
Macchione 2019 [51]	2019	Europe	Unclear risk
Maurizi 2019 [52]	2019	Europe	Unclear risk
Cai 2014 [53]	2014	Europe	Unclear risk
Nickel 2011b [54]	2011	North America	Low risk
Nickel 2004 [55]	2004	North America	Unclear risk
Pontari 2010 [56]	2012	North America	Low risk

**Table 2:** Summary of Findings and Certainty of Evidence (GRADE).

Comparison	Number of Studies (N = 56 Total)	Pooled Effect (MD, 95% CI)	Effect Magnitude & Significance	Downgrading Factors	Certainty of Evidence
Alpha-blockers vs. Placebo	22	−5.13 (−6.87 to −3.39)	Significant reduction in NIH-CPSI	Risk of Bias, Inconsistency (*I*^2^ = 98%)	Low
Analgesics vs. Placebo	7	−2.47 (−4.24 to −0.70)	Significant reduction in NIH-CPSI	Risk of Bias, Inconsistency (*I*^2^ = 88%)	Low
Antibiotics vs. Placebo	5	−2.45 (−5.53 to 0.64)	Non-significant reduction in NIH-CPSI	Risk of Bias, Inconsistency (*I*^2^ = 75%), Imprecision (CI crosses null)	Very Low
Pollen Extract vs. Placebo	9	−2.56 (−10.83 to 5.71)	Non-significant	Risk of Bias, Inconsistency (*I*^2^ = 99%), Imprecision (Wide CI/Null)	Very Low
TCM vs. Placebo	9	−3.14 (−5.38 to −0.90)	Significant reduction in NIH-CPSI	Risk of Bias, Inconsistency (*I*^2^ = 82%)	Low
Other Medications vs. Placebo	5	−6.94 (−19.79 to 5.91)	Non-significant	Risk of Bias, Inconsistency (*I*^2^ = 96%), Imprecision (Wide CI/Null)	Very Low

## Data Availability

All data generated or analyzed during this study are included in the published article [and its Supplementary Information Files]. Data used to generate forest plot is in Supplementary Table S3.

## Supplementary Materials

The following supporting information can be downloaded at: https://www.mdpi.com/article/10.3390/healthcare13222956/s1, Table S1: Search strategies used in each database; Table S2: Characteristics of randomized controlled trials included in the systematic review and meta-analysis (n = 56); Table S3: Data for forest plot; Figure S1: Forest plot showing the effect of alpha-blockers on NIH-CPSI total scores in men with chronic prostatitis/chronic pelvic pain syndrome (CP/CPPS) by risk of bias (High, Low, and Unclear). Negative MD indicate greater symptom improvement compared with placebo; Figure S2: Forest plot showing the effect of alpha-blockers on NIH-CPSI total scores in men with chronic prostatitis/chronic pelvic pain syndrome (CP/CPPS) by study setting (Western vs. Asian). Negative MD indicate greater symptom improvement compared with placebo.

## Abbreviations

The following abbreviations are used in this manuscript:

IQR	Interquartile range
g	grams
mg	Milligram
ml	Milliliter
IU	International units
BID	bis in die (twice a day)
qd	quaque die (every day)
NSAIDs	Nonsteroidal Anti-Inflammatory Drugs
NR	Not reported
TCM	Traditional Chinese Medicine
SD	Standard Deviation
