# Supplementary material for: Comparative Efficacy of Pharmacological Interventions for Chronic Prostatitis/Chronic Pelvic Pain Syndrome: An Updated Systematic Review and Meta-Analysis of Randomized Controlled Trials
Source: Healthcare (Basel). 2025 Nov 18;13(22):2956. doi: 10.3390/healthcare13222956 (PMC12652971; doi:10.3390/healthcare13222956)
Supplement: Supplementary file 1 [file healthcare-13-02956-s001.zip › healthcare-3831423-supplementary.pdf]

Supplemental Table S1. Search strategies used in each database

Database	Search String
**PubMed**	(“chronic prostatitis”[Title/Abstract] OR “chronic pelvic pain syndrome”[Title/Abstract] OR “CP/CPPS”[Title/Abstract]) AND (“alpha blocker*”[Title/Abstract] OR terazosin OR doxazosin OR phenoxybenzamine OR tamsulosin OR alfuzosin OR silodosin OR antibiotic* OR ciprofloxacin OR levofloxacin OR finasteride OR NSAID* OR corticosteroid* OR antileukotriene* OR tiocolchicoside OR pollen OR phytotherapy OR flavonoid* OR cranberr* OR “Traditional Chinese Medicine” OR TCM OR “botulinum toxin” OR anticonvulsant* OR “5-alpha reductase inhibitor*”) AND (“randomized controlled trial”[Publication Type] OR “randomized”[Title/Abstract] OR “RCT”[Title/Abstract])
**Scopus**	TITLE-ABS-KEY((“chronic prostatitis” OR “chronic pelvic pain syndrome” OR CP/CPPS) AND (“alpha blocker*” OR terazosin OR doxazosin OR phenoxybenzamine OR tamsulosin OR alfuzosin OR silodosin OR antibiotic* OR ciprofloxacin OR levofloxacin OR finasteride OR NSAID* OR corticosteroid* OR antileukotriene* OR tiocolchicoside OR pollen OR phytotherapy OR flavonoid* OR cranberr* OR “Traditional Chinese Medicine” OR TCM OR “botulinum toxin” OR anticonvulsant* OR “5-alpha reductase inhibitor*”) AND (“randomized controlled trial” OR randomized OR RCT))
**ScienceDirect**	(“chronic prostatitis” OR “chronic pelvic pain syndrome” OR CP/CPPS) AND (“alpha blocker*” OR terazosin OR doxazosin OR phenoxybenzamine OR tamsulosin OR alfuzosin OR silodosin OR antibiotic* OR ciprofloxacin OR levofloxacin OR finasteride OR NSAID* OR corticosteroid* OR antileukotriene* OR tiocolchicoside OR pollen OR phytotherapy OR flavonoid* OR cranberr* OR “Traditional Chinese Medicine” OR TCM OR “botulinum toxin” OR anticonvulsant* OR “5-alpha reductase inhibitor*”) AND (randomized OR “randomized controlled trial” OR RCT)
**Google Scholar**	“chronic prostatitis” OR “chronic pelvic pain syndrome” AND pharmacological treatment AND randomized trial. (Note: Google Scholar was used only for citation chasing and

Database	Search String
	supplementary identification of potentially relevant articles, not as a primary systematic source.)

Supplemental Table S2: Characteristics of randomized controlled trials included in the systematic review and meta-analysis (n = 56).

Study	Intervention	Control	Intervention Dose	Intervention group	Age intervention	Age control	Follow-up
Cheah 2003[26]	Terazosin	Placebo	Terazosin 1 mg for 4 days, 2 mg for 10 days and then 5 mg	Alpha-blocker	36 (24–49)	35 (20–50)	12 weeks
Erdemir 2010[27]	Terazosin	Placebo	Terazosin 5 mg	Alpha-blocker	34.76 ± 7.99	33.72 ± 8.25	12 weeks
Jung 2006[28]	Terazosin	Placebo	Terazosin 3–4 mg/day	Alpha-blocker	NR	NR	12 weeks
Wang 2016[29]	Terazosin	Placebo	Terazosin 2 mg once daily	Alpha-blocker	22.3 (SD 6.6)	22.6 (SD 6.8)	6 weeks
Yang 2010[30]	Terazosin	Placebo	Terazosin 1 mg /day	Alpha-blocker	31.0 ± 6.6	30.1 ± 6.2	12 weeks
Kulovac 2007[31]	Doxazosin	Placebo	Doxazosin 2 mg	Alpha-blocker	Mean 40.3 (SD 8.23)		6 weeks
TuAYcu 2007[32]	Doxazosin	Placebo	Doxazosin 4 mg a day	Alpha-blocker	Mean 29.1 (SD 5.2)		6 months
Wu 2008[33]	Doxazosin	Placebo	Doxazosin 4 mg a day	Alpha-blocker	34.5 ± 8.5	35.0 ± 8.8	12 weeks

Youn 2008[34]	Doxazosin	Placebo	Dose was not defined	Alpha-blocker	41.3 ± 9.5	41.8 ± 9.2	6 weeks
Lu 2004[35]	Phenoxybenzamine	Placebo	10 mg twice a day	Alpha-blocker	39.8 ± 4.5	39.1 ± 4.3	1 month
Alexander 2004[36]	Tamsulosin	Placebo	Tamsulosin 0.4 mg	Alpha-blocker	45.3 (SD 9.7)	42.6 (SD 12.0)	6 weeks
Chen 2011[37]	Tamsulosin	Placebo	Tamsulosin 0.2 mg/day	Alpha-blocker	35.3 ± 6.8	33.3 ± 7.2	6 months
Kim 2003[38]	Tamsulosin	Placebo	Tamsulosin 0.2 mg/day	Alpha-blocker	NR	NR	8 weeks
Yang 2010[30]	Tamsulosin	Placebo	Tamsulosin 1 mg/day	Alpha-blocker	31.1 ± 6.4	30.1 ± 6.2	12 weeks
Mehik 2003[39]	Alfuzosin	Placebo	Alfuzosin 5 mg twice daily	Alpha-blocker	49	50	6 months
Mo 2006[40]	Alfuzosin	Placebo	Alfusozin 10 mg/ day	Alpha-blocker	44.7 ± 7.5	45.8 ± 9.6	2 mo
Nickel 2008[7]	Alfuzosin	Placebo	Alfusozin 10 mg/ day	Alpha-blocker	40.1 (SD 12.3)	40.1 (SD 11.4)	12 weeks
Ryu 2007[41]	Alfuzosin	Placebo	Alfusozin 10 mg/ day	Alpha-blocker	41.6 ± 9.2	38.5 ± 7.8	2 months
Nickel 2011a[42]	Silodosin	Placebo	Silodosin 4 mg daily	Alpha-blocker	49.2 (SD 13.3)	49 (SD 11.6)	12 weeks
Sivkov 2005[43]	Terazosin	Placebo	Terazosin 1 to 5 mg/day	Alpha-blocker	NR	NR	8 weeks
Chen 2011[37]	Tamsulosin	Placebo	Tamsulosin 0.2 mg/day	Alpha-blocker	35.3 ± 6.8	33.3 ± 7.2	30 months
Alexander 2004[36]	Ciprofloxacin	Control	Ciprofloxacin 500 mg, 1 tablet, twice daily	Antibiotics	45.9 (SD 11.7)	42.6 (SD 12.0)	6 weeks
Kim 2011a[44]	Ciprofloxacin	Control	500 mg of Ciprofloxacin twice daily	Antibiotics	46.1	45.7	12 weeks
Kulovac 2007[31]	Ciprofloxacin	Control	Ciprofloxacin 2 doses of 500 mg daily	Antibiotics	Mean 40.3 (SD 8.23)		1 month

Nickel 2003a[45]	Levofloxacin	Control	Levofloxacin 500 mg a day	Antibiotics	56 (39 to 77)	56.2 (36 to 78)	6 weeks
Wang 2016[29]	Levofloxacin	Control	Levofloxacin 200 mg twice daily	Antibiotics	36.6 (SD 8.1)	38.5 (SD 8.3)	6 weeks
Yang 2009[30]	Corticosteroids	Control	Prednisone acetate 15 mg / day	Analgesic	29. 4 ±7.7	29.1 ± 7.0	4 weeks
Jiang 2009[46]	NSAIDs	Control	Indomethacin 25 mg 3 times a day	Analgesic	33.56 (SD 5.61)	32.23 (SD 4.41)	4 weeks
Kim 2003[38]	NSAIDs	Control	Ibuprofen 600mg tid po	Analgesic	NR	NR	8 weeks
Kim 2011a[44]	NSAIDs	Control	50 mg of diclofenac twice daily	Analgesic	46.1	45.7	12 weeks
Wu 2008[33]	NSAIDs	Control	Diclofenac 75 mg/day	Analgesic	34.5 ± 8.5	35.0 ± 8.8	12 weeks
Zhao 2009[47]	NSAIDs	Control	Celecoxib 200 mg daily	Analgesic	NR	NR	8 weeks
TuAYcu 2007[32]	Thiocolchicoside and ibuprofen	Control	Ibuprofen 400 mg and thiocolchicoside 12 mg daily	Analgesic	Mean 29.1 (SD 5.2)		6 months
Falahatkar 2015[48]	Botulinum Toxin A	Control/Saline	100 IU of Botulinum Toxin A	Botulinum Toxin A	42.67 (SD 11.24)	38.17 (SD 11.77)	4 weeks
Gottsch 2011[49]	Botulinum Toxin A	Control/Saline	Onabotulinum toxin A	Botulinum Toxin A	47 (25–77)	54 (25–80)	8 weeks
Li 2003[50]	Prostant	Control	QianLieAnShuan (Prostat) every night	TCM	20 ~ 50; mean 32.7		4 weeks
Tan 2009[51]	Prostant	Control	QianLie AnShuan (Prostat) 2 g once a day	TCM	33.56 ± 8.43	33.00 ± 8.46	6 weeks
Li 2012[50]	Qianlieantong	Control	Qianlieping capsule (2.0 g) 3 times a day	TCM	18 ~ 42, mean ± SD: 30.6 ± 6.4		6 weeks
Sun 2008[52]	Qianlieantong	Control	QianLieAnTong tablet (0.38 g per tablet)	TCM	24.46 (SD 5.38)	23.51 (SD 4.86)	4 weeks

Xia 2014[53]	Yuleshu	Control	YuLeShu Oral Mixture (20mL) 3 times a day	TCM	33.88 ± 5.68	34.54 ± 6.45	4 weeks
Zhang 2007[54]	Aike Mixture	Control	Aike decoction twice a day, 1 pack each time	TCM	32.42 ± 7.29	30.79 ± 7.64	4 weeks
Hu 2015[55]	Bazhengsan	Control	Bazheng decoction. 320mL daily	TCM	32.4 ± 5.8	31.9 ± 5.5	2 weeks
Zhang 2007[54]	Bazhengsan	Control	Bazhengsan decoction BID, 1 pack each time	TCM	20.98 ± 7.27	31.9 ± 5.5	2 weeks
Zhang 2007[54]	Decoction	Control	decoction twice a day, 1 pack each time	TCM	21.61 ± 6.69	31.9 ± 5.5	2 weeks
Wagenlehner 2009[56]	Pollen extract	Other Agents	Two capsules every 8 h, with the active substance consisting of 60 mg Cernitin T60 (water soluble fraction) and 3 mg Cernitin GBX (fat soluble fraction)	Pollen Extract	39.7 ± 7.2	39.3 ± 9.1	12 weeks
Breusov 2014[57]	Prolit Super Septo	Other Agents	Prolit Super, 2 capsules at breakfast and lunchtime orally for 2 months, containing: a tablet of 600 mg strobilanthi folium, orthosiphonis folium, radix ginseng, sea horse, imperatae rhizoma, glycyrrhizae radix	Pollen Extract	NR	NR	8 weeks

Morgia 2017[58]	Calendula-Curcuma	Other Agents	Rectal suppositories of curcumin extract 350 mg (95%) and calendula extract 80 mg, 1 suppository daily	Pollen Extract	32 (IQR 29–38)	32 (IQR 28.75–38.75)	4 weeks
Shoskes 1999[59]	Quercetin	Other Agents	Quercetin capsules 500 mg orally twice daily	Pollen Extract	43.5 (SD 3.7)	46.2 (SD 4)	4 weeks
Park 2005[60]	Cranberry	Other Agents	Cranberry Juice (Ocean Spray®) 150 mL BID	Pollen Extract	36.2 (24–45)	35.2 (23–47)	12 weeks
Cai 2017[61]	Deprox	Other Agents	Deprox 500 ® 2 tablets (Pollen extract); bromelain 80mg	Pollen Extract	32.4 ± 4.3	32.8 ± 4.9	12 weeks
Macchione 2019[62]	Deprox	Other Agents	Deprox 500® bid; Serenoa repens 320 mg	Pollen Extract	42.7 ± 11.6	39.9 ± 11.6	6 weeks
Maurizi 2019[63]	Deprox	Other Agents	DEPROX 500® 2 capsules; quercetin 1000mg	Pollen Extract	34 ± 5.9	33.7 ± 4.62	4 weeks
Cai 2014[64]	Deprox	Other Agents	Deprox 500® 2 capsules; ibuprofen 1800mg	Pollen Extract	33.8 ± 6.78	33.7 ± 5.44	4 weeks
Nickel 2011a[65]	Dutasteride	Placebo	dutasteride 0.5mg; placebo 0.5mg	5-alpha reductase inhibitors	NR	NR	192 weeks
Nickel 2004[66]	Finasteride	Placebo	finasteride 5mg qd; placebo 5mg	5-alpha reductase inhibitors	46.9 ± 1.7	41.7 ± 2.1	6 weeks

Pontari 2010[67]	Pregablin	Placebo	Pregabalinand placebo (150mg/d for 2weeks, 300mg/d for 2 weeks, 600mg/d for 2 weeks	Anticonvulsants	48.0 ± 13.0	45.2 ± 12.2	6 weeks

IQR: Interquartile range; g: grams; mg: Milligram; ml: Milliliter; IU: International units; BID: bis in die (twice a day); qd: quaque die (every day); NSAIDs: Nonsteroidal Anti-Inflammatory Drugs; NR: Not reported; TCM: Traditional Chinese Medicine; SD: Standard Deviation.

Supplemental Table S3: Data for forest plot

ID	studlab	year	Group	Med	label.e	label.c	n.e	n.c	mean.e	sd.e	mean.c	sd.c
1	Cheah 2003	2004	Alpha	Terazosin	Alpha-blocker	Placebo	43	43	10.8	9	17	12.1
2	Erdemir 2010	2010	Alpha	Terazosin	Alpha-blocker	Placebo	45	25	12.2	5.9	14.31	3.9
3	Jung 2006	2006	Alpha	Terazosin	Alpha-blocker	Placebo	71	56	14.2	6.2	19.5	6.3
4	Wang 2016	2016	Alpha	Terazosin	Alpha-blocker	Placebo	39	38	11.369	4.125	13.263	4.346
5	Yang 2010	2010	Alpha	Terazosin	Alpha-blocker	Placebo	51	25	11.1	1.62	24.14	3.31
6	Kulovac 2007	2007	Alpha	Doxazosin	Alpha-blocker	Placebo	30	30	15.867	5.78	20.8	4.27
7	TuAYcu 2007	2007	Alpha	Doxazosin	Alpha-blocker	Placebo	29	28	10.7	1.3	21.9	1.2
8	Wu 2008	2008	Alpha	Doxazosin	Alpha-blocker	Placebo	39	37	16.77	1.37	18.14	1.69
9	Youn 2008	2008	Alpha	Doxazosin	Alpha-blocker	Placebo	34	35	13.4	5.3	16.5	5.4
10	Lu 2004	2004	Alpha	Phenoxybenzamine	Alpha-blocker	Placebo	20	20	13.75	3.29	19.55	3.51
11	Alexander 2004	2004	Alpha	Tamsulosin	Alpha-blocker	Placebo	45	45	-4.4	6.3	-3.4	5
12	Chen 2011	2011	Alpha	Tamsulosin	Alpha-blocker	Placebo	50	49	14.8	4.2	18.5	3.6

13	Kim 2003	2003	Alpha	Tamsulosin	Alpha-blocker	Placebo	22	15	13.32	6.81	17.07	5.36
14	Yang 2010	2010	Alpha	Tamsulosin	Alpha-blocker	Placebo	51	25	9.24	1.9	24.14	3.31
15	Mehik 2003	2003	Alpha	Alfuzosin	Alpha-blocker	Placebo	17	20	-9.9	5.79	-3.8	7.08
16	Mo 2006	2006	Alpha	Alfuzosin	Alpha-blocker	Placebo	26	28	11	4.5	15.6	5.6
17	Nickel 2008	2008	Alpha	Alfuzosin	Alpha-blocker	Placebo	116	117	-7.1	9	-6.5	8.5
18	Ryu 2007	2007	Alpha	Alfuzosin	Alpha-blocker	Placebo	42	15	10.5	1	12.1	0.9
19	Nickel 2011a	2011	Alpha	Silodosin	Alpha-blocker	Placebo	52	51	-12.1	9.3	-8.5	7.2
20	Sivkov 2005	2005	Alpha	Terazosin	Alpha-blocker	Placebo	29	22	14.48	5.28	22.24	5.95
21	Chen 2011	2011	Alpha	Tamsulosin	Alpha-blocker	Placebo	45	47	17.3	3.1	18.7	3.4
22	Mehik 2003	2003	Alpha	Alfuzosin	Alpha-blocker	Placebo	16	19	-3.5	5.77	-0.1	5.96
23	TuAYcu 2007	2007	Alpha	Doxazosin	Alpha-blocker	Placebo	29	28	12.5	1	22.2	1.1
24	Alexander 2004	2004	Antibiotics	Ciprofloxacin	Antibiotics	Control	42	45	-6.2	7.3	-3.4	5
25	Kim 2011a	2011	Antibiotics	Ciprofloxacin	Antibiotics	Control	28	40	8.1	4.2	8.6	4.1
26	Kulovac 2007	2007	Antibiotics	Ciprofloxacin	Antibiotics	Control	30	30	15.867	5.78	18.267	3.88
27	Nickel 2003a	2003	Antibiotics	Levofloxacin	Antibiotics	Control	45	35	18.8	10.7	18.2	9.3
28	Wang 2016	2016	Antibiotics	Levofloxacin	Antibiotics	Control	39	38	11.369	4.125	17.35	5.358
29	Yang 2009	2009	Analgesic	Corticosteroids	Analgesic	Control	78	80	11.08	5.41	13.9	2.05
30	Jiang 2009	2009	Analgesic	NSAIDs	Analgesic	Control	80	35	7.145	1.936	12.8	3.23
31	Kim 2003	2003	Analgesic	NSAIDs	Analgesic	Control	22	18	13.32	6.81	12.61	5.19
32	Kim 2011a	2011	Analgesic	NSAIDs	Analgesic	Control	32	40	7.7	4.9	8.6	4.1

33	Wu 2008	2008	Analgesic	NSAIDs	Analgesic	Control	39	39	16.77	1.37	18.51	1.67
34	Zhao 2009	2009	Analgesic	NSAIDs	Analgesic	Control	32	32	15.88	2.51	19.5	2.5
35	TuAYcu 2007	2007	Analgesic	Thiocolchicoside and ibuprofen	Analgesic	Control	29	29	9.2	1	10.7	1.3
36	Falahatkar 2015	2015	Others	Botulinum Toxin A	Botulinum Toxin A	Control/ Saline	30	30	10.57	11.34	36.37	4.4
37	Gottsch 2011	2011	Others	Botulinum Toxin A	Botulinum Toxin A	Control/ Saline	13	16	-2.2	4.3	0.4	3.8
38	Li 2003	2003	TCM	Prostant	TCM	Control	41	35	9.12	5.262	11.17	5.315
39	Tan 2009	2009	TCM	Prostant	TCM	Control	45	43	7.44	6.19	13.4	7.23
40	Li 2012	2012	TCM	Qianlieantong	TCM	Control	98	56	8.2	4.1	11.6	5.4
41	Sun 2008	2008	TCM	Qianlieantong	TCM	Control	73	42	7.68	3.49	14.35	4.06
42	Xia 2014	2014	TCM	Yuleshu	TCM	Control	44	44	13.61	2.81	15.02	2.73
43	Zhang 2007	2007	TCM	Aike Mixture	TCM	Control	58	16	10.83	4.9	14.42	9.13
44	Hu 2015	2015	TCM	Bazhengsan	TCM	Control	48	48	11.92	6.85	17.14	7.32
45	Zhang 2007	2007	TCM	Bazhengsan	TCM	Control	50	16	17.13	7.65	14.42	9.13
46	Zhang 2007	2007	TCM	Decoction	TCM	Control	62	16	15.49	7.57	14.42	9.13
47	Wagenlehner 2009	2009	Pollen	Pollen extract	Pollen Extract	Other Agents	68	69	-7.66	5.77	-5.16	5.81

48	Breusov 2014	2014	Pollen	Prolit Super Septo	Pollen Extract	Other Agents	29	28	10.3	6.46	19.3	20.11
49	Morgia 2017	2017	Pollen	Calendula-Curcuma	Pollen Extract	Other Agents	24	24	14.5	2.31	20.5	2.2
50	Shoskes 1999	1999	Pollen	Quercetin	Pollen Extract	Other Agents	15	13	13	6.584	18.8	6.851
51	Park 2005	2005	Pollen	Cranberry	Pollen Extract	Other Agents	26	24	12.7	3.9	18.1	3.7
52	Cai 2017	2017	Pollen	Deprox	Pollen Extract	Other Agents	36	34	11.7	3.2	22.5	3.7
53	Macchione 2019	2019	Pollen	Deprox	Pollen Extract	Other Agents	40	43	31	3.8	6	5.3
54	Maurizi 2019	2019	Pollen	Deprox	Pollen Extract	Other Agents	27	27	12.22	1.84	14.85	1.85
55	Cai 2014	2014	Pollen	Deprox	Pollen Extract	Other Agents	41	46	12.8	2.2	19.5	2.1
56	Nickel 2011a	2011	Others	Dutasteride	5-alpha reductase inhibitors	Placebo	341	343	-5.2	6.6	-2.7	7.2
57	Nickel 2004	2004	Others	Finasteride	5-alpha reductase inhibitors	Placebo	33	31	19.1	1.5	21.7	1.6
58	Pontari 2010	2012	Others	Pregablin	Anticonvulsants	Placebo	218	106	19.7	8.5	21.6	8.9

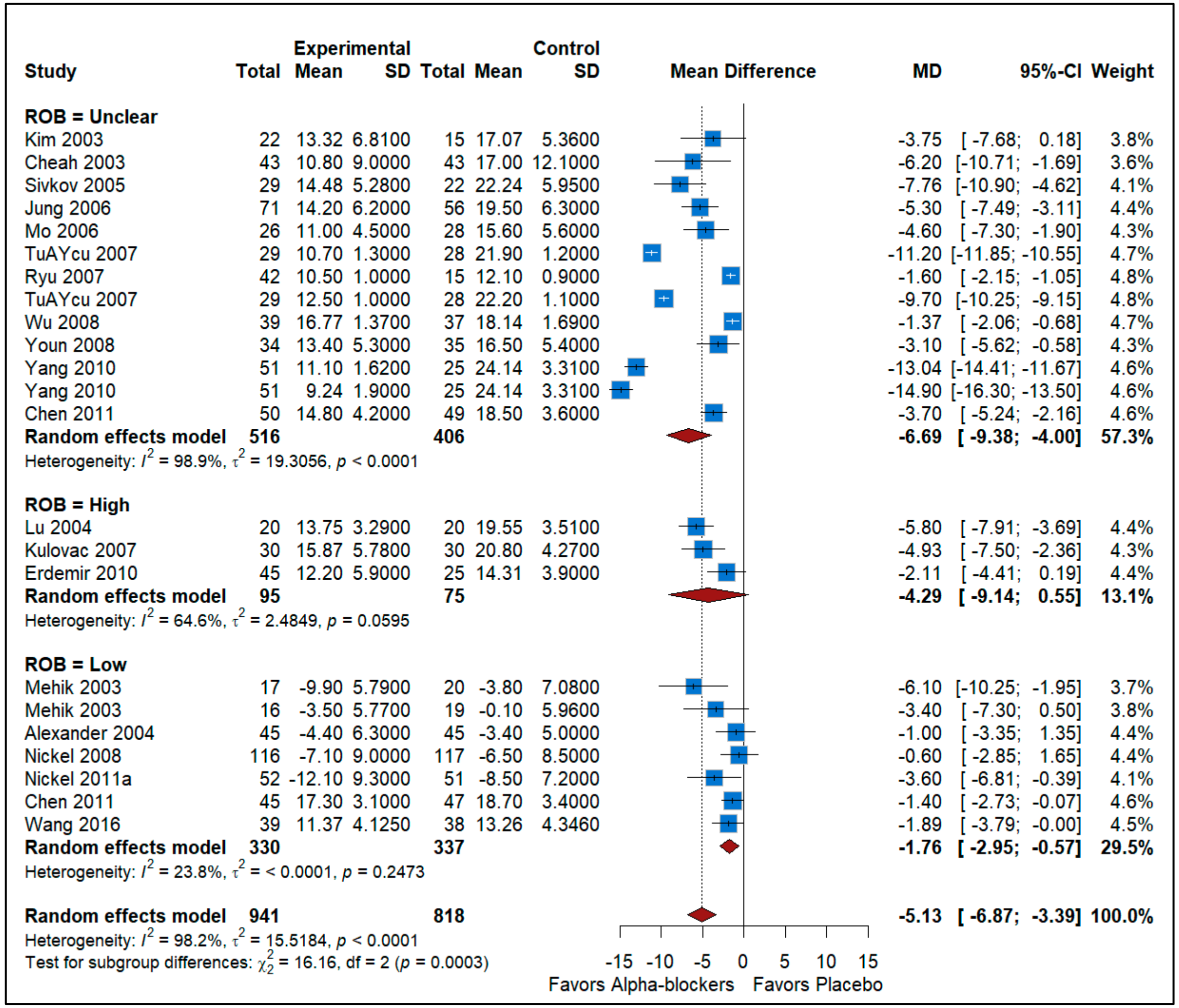

Supplemental Figure S1: Forest plot showing the effect of alpha-blockers on NIH-CPSI total scores in men with chronic prostatitis/chronic pelvic pain syndrome (CP/CPPS) by risk of bias (High, Low, and Unclear). Negative MD indicate greater symptom improvement compared with placebo.

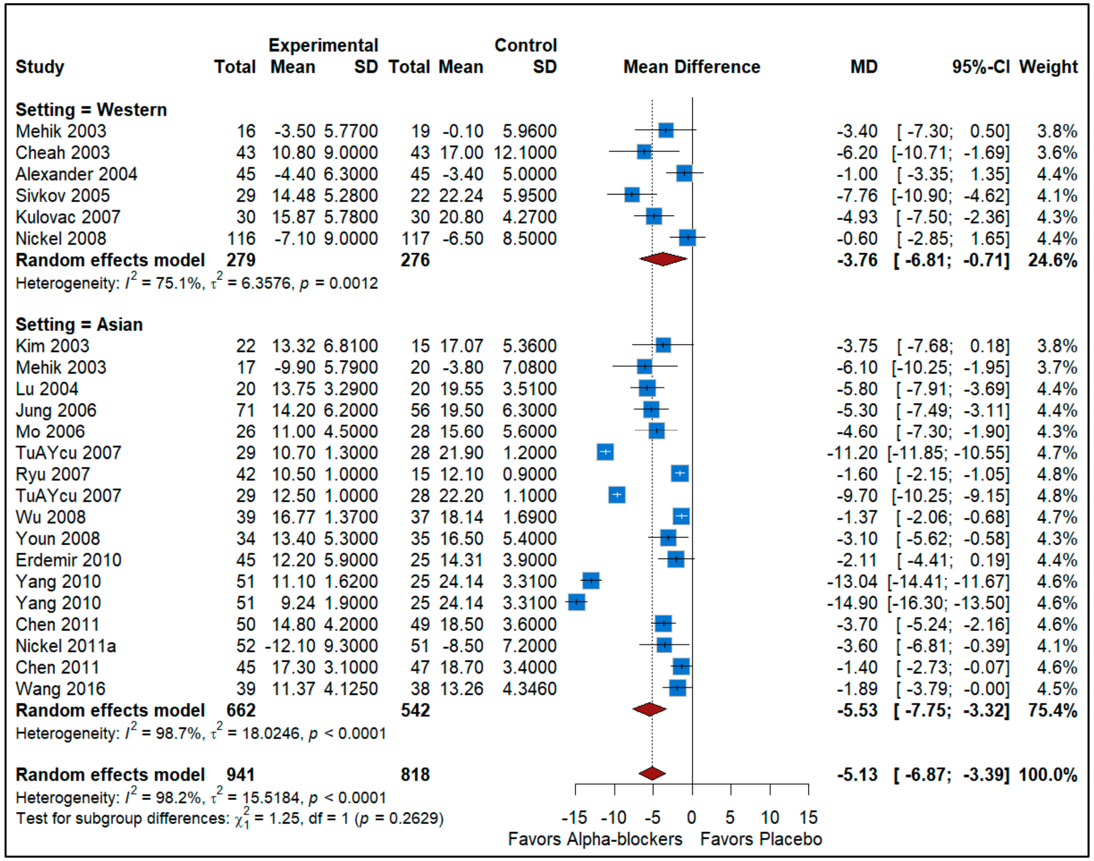

Supplemental Figure S2: Forest plot showing the effect of alpha-blockers on NIH-CPSI total scores in men with chronic prostatitis/chronic pelvic pain syndrome (CP/CPPS) by study setting (Western vs. Asian). Negative MD indicate greater symptom improvement compared with placebo.
